# Genetic parameter estimation for live weight during different life periods in Anatolian buffalo raised in Istanbul

**DOI:** 10.5194/aab-69-69-2026

**Published:** 2026-01-27

**Authors:** Kemal Yazgan, Mehmet İhsan Soysal, Yasemin Öner, Eser Kemal Gürcan, Emel Özkan Ünal

**Affiliations:** 1 Harran University, Faculty of Agriculture, Animal Science Department, 63300, Şanlıurfa, Türkiye; 2 Tekirdağ Namık Kemal University, Faculty of Agriculture, Animal Science Department, 59030, Tekirdağ, Türkiye; 3 Bursa Uludağ University, Faculty of Agriculture, Animal Science Department, 16059, Bursa, Türkiye

## Abstract

In Türkiye, as in many parts of the world, buffalo play a significant role in livestock production alongside cattle. Although buffaloes generally exhibit lower productivity than cattle, they are valued for their resilience against challenging environmental conditions and for the unique quality of their milk and meat products. This study aimed to estimate the genetic parameters of live-weight gain, a key trait for improving profitability in buffalo breeding. Heritability estimates were obtained for birth weight (BW), live weight at 6 months (LW_6_), and live weight at 12 months (LW_12_) using data from 910 animals across 42 farms in the province of Istanbul. Genetic evaluations were performed using the BUGA 1.0 software, applying the AI-REML algorithm.

The mean weights were 39.02 ± 0.169 kg (BW), 140.86 ± 0.4 kg (LW_6_), and 255.97 ± 0.692 kg (LW_12_). The corresponding heritability estimates were 0.5006 ± 0.000029 for BW, 0.5001 ± 0.000035 for LW_6_, and 0.5000 ± 0.0000012 for LW_12_. Additive genetic effects exhibited moderate to high accuracy, ranging from 0.63 to 0.68. The proportion of animals with positive additive genetic effects was relatively high for LW_6_ and LW_12_ at 49.34 % and 48.13 %, respectively. Genetic trend analysis was also conducted over time for all three traits, highlighting the potential for selection-based improvement in Anatolian buffalo.

## 1 Introduction

The domestic water buffalo (*Bubalus bubalis*) descends from various wild Asian buffalo species, primarily *Bubalus arnee*. Two main types, riverine and swamp buffaloes, belong to this species and were domesticated at different times. Due to its adaptability and unique product qualities, buffalo farming has become widespread in nearly half of the world's countries (Cockrill, 1974; FAO, 2000; Zhang et al., 2020). India, Pakistan, and China currently host the largest buffalo populations, while European countries such as Italy maintain economically valuable buffalo farming for high-quality dairy products (FAOSTAT, 2023).

The Anatolian water buffalo raised in Türkiye is a descendant of the Mediterranean buffalo, a sub-branch of the riverine type, and is believed to have entered the country in the 7th century (Anonymous, 2024a, b). According to the Turkish Statistical Institute (TURKSTAT, 2025), the buffalo population in Türkiye reached 162 051 in 2024, reflecting a slight annual increase of 0.02 %.

Although buffaloes are less productive than cattle in terms of reproductive rate and efficiency, they are highly valued for their tolerance of harsh environmental conditions and for the distinct nutritional and culinary properties of their milk and meat. These advantages have spurred growing interest in buffalo husbandry, especially given the health benefits associated with buffalo-derived products.

To enhance both the population and productivity of buffaloes in Türkiye, the Ministry of Agriculture and Forestry launched the “Anatolian Buffalo National Breeding Project” in 2011 through its Directorate General of Agricultural Research and Policies as a community-based water buffalo genetic improvement programme. Currently active in 18 provinces, the project has registered over 30 000 animals. There has been a significant rise in studies investigating genetic parameters in these populations, though most have focused on milk yield rather than growth traits. For example, a study by Öztürk et al. (2024) on carcass weights in the Thrace region reported a 46 kg increase in slaughter weight between 2017 and 2021, indicating the potential impact of genetic and management improvements.

Traits like birth weight (BW), live weight at 6 months (LW_6_), and at 12 months (LW_12_) are essential indicators of growth performance and are closely linked to age at puberty, lifetime productivity, and general health status. Estimating genetic parameters for these traits is crucial for effective breeding and selection strategies. Research on growth-related traits such as live weight remains limited. Pandya et al. (2015) reported heritability values of 0.188 ± 0.112, 0.216 ± 0.122, and 0.144 ± 0.096 for BW, LW6, and LW12, respectively. In addition, Bolívar et al. (2013) showed that the heritability for live weight ranged from 0.32 to 0.18 from birth to 1 year of age and then increased to 0.39. Also, heritability values for BW have been reported to be 0.190 by El-Sayed et al. (2020), 0.26 ± 0.036 by Zaghloul et al. (2024), 0.35 ± 0.03 by El-Awady et al. (2005), and 0.41 by Salem et al. (2021).

This study aimed to estimate the heritability of BW, LW_6_, and LW_12_ in Anatolian buffalo raised under field conditions in the Istanbul region. The data were obtained from 910 animals raised on 42 farms participating in the national breeding programme. Genetic evaluations were conducted using the AI-REML algorithm implemented in BUGA software, which was developed to meet Türkiye's national needs with regard to livestock genetics.

## 2 Material and methods

In Türkiye, as in many parts of the world, buffalo play a significant role in livestock production alongside cattle. Although buffaloes generally exhibit lower productivity than cattle, they are valued for their resilience against challenging environmental conditions and for the unique quality of their milk and meat products. This study aimed to estimate the genetic parameters of live-weight gain, a key trait for improving profitability in buffalo breeding. Heritability estimates were obtained for birth weight (BW), live weight at 6 months (LW_6_), and live weight at 12 months (LW_12_) using data from 910 animals across 42 farms in the province of Istanbul. Genetic evaluations were performed using the BUGA 1.0 software, applying the AI-REML algorithm.

Genetic trend analysis was also conducted over time for all three traits, highlighting the potential for selection-based improvement in Anatolian buffalo.

To estimate genetic parameters and variance components, data on calf weight gain from the Anatolian Buffalo National Breeding Project were retrieved from the Manda Yıldızı database (Tekerli, 2019). The birth weights of 910 calves born between 2012 and 2024 were collected from 42 farms located in various districts of Istanbul, including Arnavutköy, Eyüp, Çatalca, and Silivri.

In the model for BW (Eq. 1), fixed effects included birth year, birth month, farm, and sex. Due to the absence of pedigree records, animals were assumed to be unrelated. For the analyses of LW_6_ and LW_12_, the same fixed effects were maintained, and birth weight was added as a covariate (Eq. 2). As the effect of LW_6_ on LW_12_ was found to be negative in the variance analysis, it was excluded from the model, and only BW was retained as a covariate.

1Yijklm=μ+hi+brj+mk+sl+eijklm2Yijklm=μ+hi+brj+mk+sl+byxXijklm+eijklm

In the above, $Y_{ijklm}$ is the LW_6_, LW12, or BW of any water buffalo ( m ) of herd ( h ) i , birth year (br) j , birth month ( m ) k , and sex ( s ) l ; μ is the overall mean; $h_{i}$ is the effect of herd ( i=1 , 2, 3 …42) br_*j*_ is the effect of birth year ( j=2012 , 2013, 2014 …2024); $m_{k}$ is the effect of birth month ( k=1 , 2, 3 …12); $s_{l}$ is the effect of sex ( l=1 and 2); $b_{yx}$ is the partial regression coefficient of Y with respect to X ; $X_{ijklm}$ is the birth weight of any water buffalo ( m ) of the herd ( h ) i , birth year (br) j , birth month ( m ) k , and sex ( s ) l ; and $e_{ijklm}$ is the random residual error.

Additionally, the model given below (Eq. 3) was used for the estimation of genetic parameters.

3 y=Xb+Zu+e

In the above, y is the vector of LW_6_, LW_12_, or BW; b is the vector of fixed effects (herd, birth year, birth month, and sex) and the covariate (birth weight); u is the vector of vector of additive genetic effects; e is the vector of the random residual effect; and X and Z are incidence matrices relating records to fixed and animal effects, respectively.

The analysis was performed on BUGA software that became widespread in Türkiye's conditions (Yazgan, 2023) using an AI-REML algorithm (Gilmour et al., 1995). The analysis was repeated using WOMBAT (Meyer, 2007) software to compare the results (Meyer, 2007).

## 3 Results 

The average values were calculated to be 39.02 ± 0.169, 140.86 ± 0.4, and 255.97 ± 0.692 kg for BW, LW_6_, and LW_12_ (Table 1). The heritabilities of birth weight and 6- and 12-month live weights were estimated to be 0.5006 ± 0.000029, 0.5001 ± 0.000035, and 0.5000 ± 0.000001, respectively (Table 2). The effect of birth weight on live weight at the 6th month was found to be significant ( p<0.05 ), while its effect on weight at the 12th month was not found to be significant. At the same time, the effect of dam age on growth characteristics was found to be insignificant ( p>0.50 ).

**Table 1:** Descriptive statistics for BW, LW_6_, and LW_12_.

Item	Mean ± SE	Minimum	Maximum	CV
	(kg)	(kg)	(kg)	(%)
BW	39.02 ± 0.169	30.00	52.00	13.08
LW_6_	140.86 ± 0.400	120.00	165.00	8.56
LW_12_	255.97 ± 0.692	220.00	346.94	8.15

SE denotes standard error, and CV denotes coefficient of variation.

**Figure 1:**
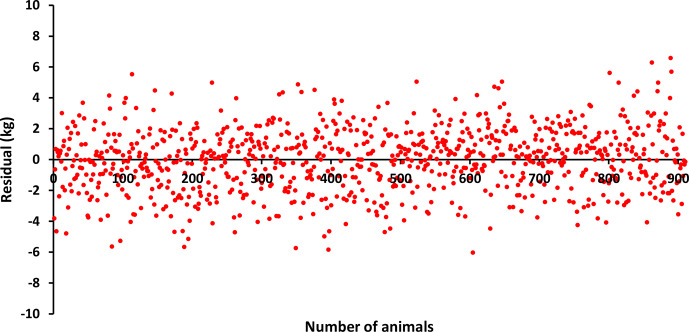
Residuals for BW.

**Figure 2:**
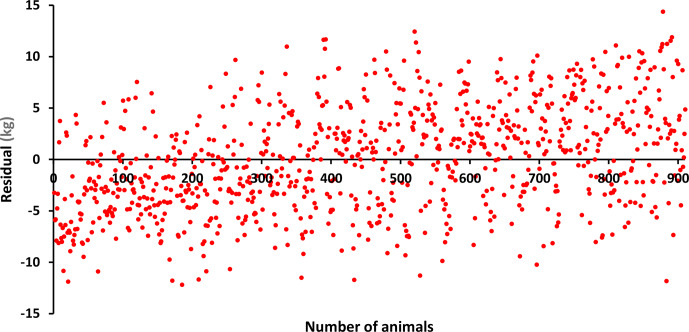
Residuals for LW_6_.

**Figure 3:**
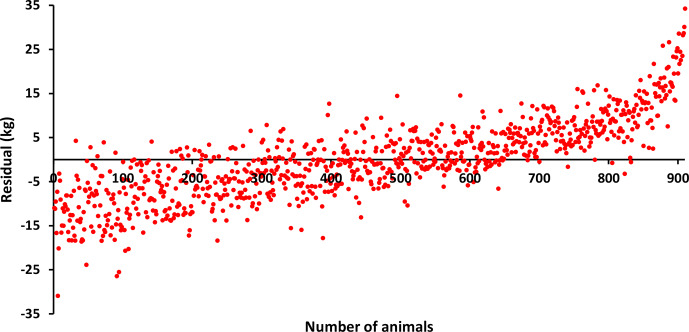
Residuals for LW_12_.

The differences (residuals) of the estimated values of BW, LW_6_, and LW_12_ obtained using the estimated b (vector of fixed effects) and u (vector of additive genetic effects) parameters in Eq. (3) from the observed values are given in Figs. 1, 2, and 3. Accordingly, when BW and LW_6_ were used as variables in the model given in Eq. (3), more homogeneous residues were obtained, while, in LW_12_, high deviations were observed at the beginning and end. This may be because the lowest MLogL value ( - 2985.15) belongs to LW_12_, as can be seen in Table 2. In other words, the animal model showed the poorest fit for LW_12_.

**Table 2:** Variance components of BW, LW_6_, and LW_12_ with standard errors.

	Variance components				
Item	Phenotypic	Genotypic	Random residual	Heritability	MLogL
BW	17.8273 ± 0.12253	8.9239 ± 1.15231	8.9033 ± 1.15231	0.5006 ± 0.000029	- 1730.94
LW_6_	112.4207 ± 0.27221	56.2185 ± 0.18262	56.2021 ± 0.18262	0.5001 ± 0.000035	- 2510.01
LW_12_	347.0691 ± 0.46246	173.5430 ± 0.05915	173.5260 ± 0.05915	0.5000 ± 0.000001	- 2985.15

BW: birth weigh; LW_6_: 6-month live weight; LW_12_: 12-month live weight; MLogL: maximum log likelihood.

Minimum and maximum additive genetic effects, along with their standard error (SE) and accuracies of prediction ( r ) for birth weight (BW), live weight at 6 months (LW_6_), and live weight at 12 months (LW_12_), are presented in Table 3. Substantial variation was observed in the additive genetic effects of 910 animals, ranging from - 6.03 to 6.60 kg for BW, from - 12.18 to 14.39 kg for LW_6_, and from - 30.89 to 37.53 kg for LW_12_. The proportion of animals showing positive additive genetic effects was high for both LW_6_ and LW_12_ at 49.34 % and 48.13 %, respectively (Table 3). These wide genetic variabilities observed for LW_6_ and LW_12_ suggest promising opportunities for improving these traits in Anatolian buffalo populations through selection.

**Table 3:** Minimum and maximum additive genetic effect, their standard error (SE), and accuracies of predictions ( r ) for BW, LW_6_, and LW_12_.

Item	Number of	Minimum	SE	r	Maximum	SE	r	Positive
	animals	additive		additive				additive
		genetic		genetic				genetic
		effect (kg)		effect (kg)				effect (%)
BW	911	- 6.03	2.147	0.69	6.60	2.314	0.63	52.52
LW_6_	911	- 12.18	5.786	0.63	14.39	5.515	0.67	49.34
LW_12_	911	- 30.89	9.507	0.69	37.53	9.556	0.68	48.13

BW: Birth weight; LW_6_: 6-month live weight; LW_12_: 12-month live weight.

For all traits, the minimum and maximum additive genetic effects were estimated with moderate to high accuracy ( r ), ranging from 0.63 to 0.68 (Table 3).

**Figure 4:**
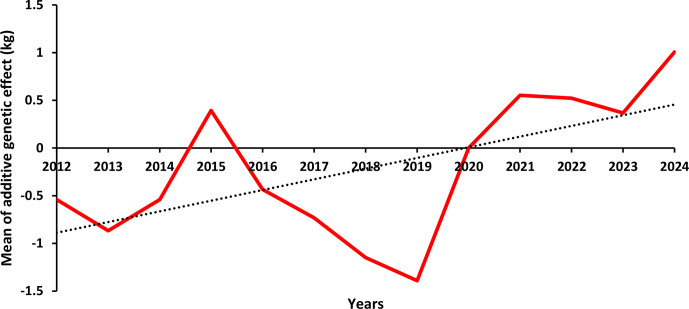
LW_6_'s average additive genetic effects by year.

**Figure 5:**
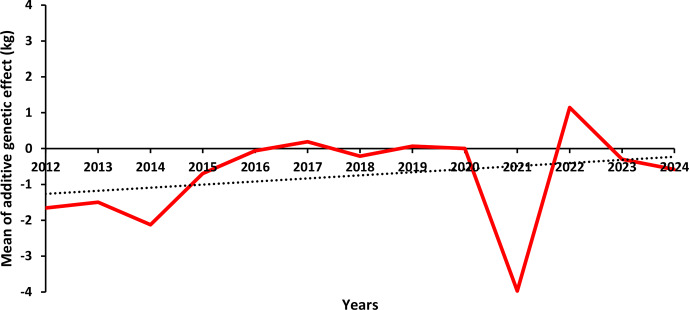
LW_12_'s average additive genetic effects by year.

Figures 4 and 5 illustrate the genetic trends for BW, LW_6_, and LW_12_ between 2012 and 2024. The annual averages of additive genetic effects for BW display a fluctuating pattern. As shown in Fig. 4, the lowest average additive genetic effect was observed between 2016 and 2018 ( - 0.53 kg), while the highest values were recorded in 2014 and 2021 (0.29 and 0.36 kg, respectively).

A similar pattern was observed for LW_6_ (Fig. 4), although a slight increasing trend was noted in recent years. The average additive genetic effect reached approximately 1.00 kg in 2024, up from 0.55 kg in 2021. In contrast, the trend for LW_12_ (Fig. 5) remained close to zero throughout the studied period, except in 2021, where it dropped to - 3.97 kg.

One of the most noteworthy findings of this study is that the data analysed using BUGA software – developed by Yazgan (2023), featuring a Turkish interface, and designed to be user-friendly under local farming conditions – yielded identical results to those obtained using the well-established WOMBAT software (Meyer, 2007).

## 4 Discussion

All of these values for the average weight for different ages are higher than those found in studies conducted in different buffalo populations from different countries, such as Italian (Rosati and Van Vleck, 2002), Egyptian (Khattab et al., 2003; El-Awady et al., 2005; Salem et al., 2021), Indian (Pandya et al., 2015), Colombian (Bolívar et al., 2013; Agudelo-Gómez et al., 2015), Brazilian (Tonhati et al., 2000), Malaysian (Soh et al., 2020), Syrian (El-Sayed et al., 2020), and Anatolian buffalos (Güllüce and Şahin, 2025). Although the average birth weight was found to be the same in Italian buffalos, the average live weights at 6 and 12 months were reported to be well below the averages estimated in our study (Rosati and Van Vleck, 2002). In studies carried out between 1970 and 2025 to evaluate growth traits in Anatolian buffalos, lower birth weights ranging between 26.50–32.30 were reported (Uslu, 1970; İzgi et al., 1989; Yılmaz et al., 2016; Erdoğan et al., 2021; Alkoyak and Öz, 2022; Kaplan and Tekerli, 2023; Güllüce, 2023; Akçam and Kul, 2025). The reason why the live-weight averages of different periods as estimated in this study differ from those previously reported for Anatolian buffalos may be that the study material was obtained from the records obtained from breeders in Istanbul. It is known that the physical and material opportunities of Istanbul and its surroundings are greater than in other geographical regions where buffalo breeding is carried out in Türkiye, and the producer profile is also different. It is particularly notable for the large number of animals per farm and its proximity to major consumption centres.

In the current study, Anatolian buffalo herds were not exposed to intensive selection procedures. Thus, in the buffalo populations investigated in the study, there is an opportunity for successful direct selection based on body weights and growth in the future. The estimated heritabilities for all three traits were also higher than those of previous studies (El-Awady et al., 2005; Bolívar et al., 2013; Pandya et al., 2015; El-Sayed et al., 2020; Salem et al., 2021; Zaghloul et al., 2024). These values were quite similar to the values estimated in study carried out by Kaplan and Tekerli (2023), except for heritability of birth weight, which was estimated to be 0.28. On the other hand, these are considerably higher than the values obtained from different geographical populations within the same project (Güllüce, 2023). The author estimated the heritabilities to be 0.24, 0.16, and 0.15 for BW, LW_6_, and LW_12_, respectively. Heritability is known to be a parameter that varies from population to population. The estimated heritability levels of the same character in previous studies conducted on Anatolian buffalos are so different due to rearing under different environmental conditions or due to the different mathematical models used in the estimation. Lack of pedigree records may be considered to be the study's main limitation. This situation may relate to the relatively high values of the heritability. Nevertheless, in most countries, it is difficult to obtain well-arranged pedigree records for ideal estimation. The high heritability estimates for all three traits evaluated can indicate that the mathematical model used may be appropriate for use in breeding value estimation.

It is not possible to measure all factors which may be influential with regard to quantitative characters. Numerous environmental factors may interact; moreover, the genetic structure of the herds may cause significant differences whose effects cannot be estimated. For example, in a recent study, the effect of an single nucleotide polymorphism (SNP) located on the growth hormone receptor gene (GHR) region was found to be significant for LW_12_ (Erdoğan et al., 2021).

Similar patterns of wide variability in predicted breeding values (PBVs) have been reported in previous studies. For instance, El-Awady et al. (2005) found PBVs ranging from - 4.8 to 3.4 kg for BW in calves. Likewise, Zaghloul et al. (2024) reported a PBV range of - 4.2 to 3.5 kg for BW. In contrast, studies by El-Sayed et al. (2020) and Salem et al. (2021) observed narrower PBV ranges for BW, from - 0.02 to 0.2 kg.

For all traits, the minimum and maximum additive genetic effects were estimated with moderate to high accuracy ( r ), ranging from 0.63 to 0.68 (Table 3). Zaghloul et al. (2024) reported similar accuracy levels for BW. The accuracy values observed in the present study suggest that calves in these herds could be reliably selected as future parents, thereby contributing to long-term genetic improvement in the growth traits of Anatolian buffalo.

The absence of a consistent upward genetic trend over the 13-year period across all traits can be attributed to several factors: (1) the limited number of herds and minimal use of inbreeding practices, (2) the lack of comprehensive pedigree records, (3) the dependence on a small number of elite sires and insufficient accuracy in performance recording, (4) the inadequate implementation of progeny testing, (5) the selection of young calves based solely on growth performance rather than estimated breeding values (EBVs), and (6) the lack of effective breeding strategies and selection methods to drive intended genetic improvement over time.

This consistency reinforces confidence in the applicability of BUGA under Turkish conditions. The software had previously demonstrated reliability in various evaluations involving Anatolian buffalo, providing consistent outcomes (Yazgan, 2023; Yazgan and Soysal, 2023; Yazgan et al., 2024). These findings suggest that BUGA can be confidently employed for genetic evaluations in national livestock improvement programmes.

## 5 Conclusions

Live-weight gain is one of the primary factors determining growth performance in buffalo, directly influencing economic efficiency and several productivity-related traits. Additionally, it reflects the general health and well-being of the animal. In the existing literature, the number of studies estimating genetic parameters for growth traits in buffalo remains limited compared to those focusing on milk yield. However, with increasing awareness of the gastronomic and health benefits of buffalo meat, the importance of growth traits is expected to rise.

This study has contributed valuable data that can support buffalo breeding initiatives in Türkiye. The relatively high heritability estimates found in this research suggest a strong potential for selection despite the lack of pedigree records. The absence of sire identification data is a major constraint for more advanced genetic modelling; however, the consistency and strength of the results underscore the robustness of the methodology. To enhance the precision and efficiency of future breeding programmes, it is crucial to maintain accessible and well-organized pedigree information. The findings highlight that, even under field conditions and with limited pedigree data, significant genetic progress can be achieved for live-weight traits in Anatolian buffalo populations in Istanbul.

## Data Availability

The data that support the findings of this study are available from the corresponding author upon reasonable request.
